# Recommendations for application of *Haemophilus influenzae* PCR diagnostics to respiratory specimens for children living in northern Australia: a retrospective re-analysis

**DOI:** 10.1186/s13104-018-3429-z

**Published:** 2018-05-21

**Authors:** Jemima Beissbarth, Michael J. Binks, Robyn L. Marsh, Anne B. Chang, Amanda J. Leach, Heidi C. Smith-Vaughan

**Affiliations:** 1 Menzies School of Health Research, Charles Darwin University, PO Box 41096, Casuarina, NT 0811 Australia; 2 Department of Respiratory Medicine, Lady Cilento Children’s Hospital, PO Box 3474, South Brisbane, QLD 4101 Australia; 3 Institute of Health and Biomedical Innovation, Queensland University of Technology, GPO Box 2434, Brisbane, QLD 4001 Australia

**Keywords:** *Haemophilus haemolyticus* (Hh), Nontypeable *Haemophilus influenzae* (NTHi), Nasopharynx, bronchoalveolar lavage

## Abstract

**Objective:**

*Haemophilus haemolyticus* can be misidentified as nontypeable *Haemophilus influenzae* (NTHi) due to their phenotypic similarities in microbiological culture. This study aimed to determine the prevalence of misidentified NTHi in respiratory specimens from children living in northern Australia.

**Results:**

Among respiratory specimens collected in studies between 2010 and 2013, retrospective PCR analysis found that routine culture misidentified *H. haemolyticus* as NTHi in 0.3% (3/879) of nasal specimens, 25% (14/55) of bronchoalveolar lavage and 40% (12/30) of throat specimens. Therefore, in this population, PCR-based NTHi diagnostics are indicated for throat and bronchoalveolar specimens, but not for nasal specimens.

## Introduction

Nontypeable *Haemophilus influenzae* (NTHi) disease represents a major global health burden [1]. NTHi are among the most common pathogens identified in the lower airways of patients with chronic lung disease; for example, chronic obstructive pulmonary disease in which up to 90% of acute exacerbations are associated with NTHi [1]. NTHi are also commonly found in children with otitis media (55–90%), bacterial conjunctivitis (44–68%) and 41% bacterial sinusitis (41%) [1]. In remote areas of northern Australia, recent cross-sectional data show NTHi in the nasopharynx of 70% young Indigenous children [2]. In this region, NTHi is the dominant pathogen cultured from the middle ear of children with tympanic membrane perforation [3], and the lungs of children with bronchiectasis [4]. By culture, NTHi from these sites are phenotypically indistinguishable from non-haemolytic strains of the typically commensal *H. haemolyticus* (Hh) [5, 6]. DNA-based methods are required to accurately distinguish NTHi from Hh, and several retrospective studies of presumptive NTHi isolates reported varying rates (0–27%) of reclassification of NTHi as Hh [6–8]. Whilst identifying Hh as a bacteria associated with an infection may not change immediate clinical treatment, understanding the proportion of misidentified NTHi in respiratory samples is important to accurately describe the burden of respiratory disease attributable to NTHi and potentially Hh.

Our aim was to determine the prevalence of misidentified NTHi in 773 specimens from the nasopharynx or nose, ear discharge, throat, and lower airways from 665 children (< 10 years of age) in northern Australia, and to determine the utility of NTHi PCR diagnostics for our future studies.

## Main text

### Methods

#### Choice of specimens tested

Our collection of presumptive NTHi isolates (stored at −80 °C) were originally cultured from nasopharyngeal or nasal (Np), ear discharge (ED), throat, and bronchoalveolar lavage (BAL) specimens collected from children < 10 years of age living in the north of Australia. All children had participated in previous (2010–2013) cross-sectional studies [2, 4, 9] or a randomised controlled trial [10] (Table 1). The studies providing isolates were purposefully chosen to ensure the isolates from the existing NTHi collection represented a broad age range (0–10 years). Isolates were restricted to collection years 2010–2013 to reduce time dependent bias. In the original studies, culture was performed using standard methods as previously described [3]. Briefly, up to four presumptive NTHi isolates from each specimen were selected on the basis of colony morphology (greyish, transparent, smooth colonies) on chocolate agar and bacitracin–vancomycin–clindamycin–chocolate agar. The isolates were confirmed as presumptive NTHi if hemin and nicotinamide adenine dinucleotide (X and V factor) dependent (Oxoid) and coagglutination negative [Haemophilus Phadebact (10557512, Remel)]. From 773 NTHi-positive specimens (Table 1), 985 isolates were further scrutinised. Of these 773 specimens, 179 (23%) contributed multiple isolates (between 2 and 4, totalling 391 isolates). Inclusion criteria: specimens collected between 2010 and 2013 in the studies outlined in Table 1 where the participant was of north Australian residence and specimens had at least one presumptive NTHi isolate. No other exclusion criteria were applied. Due to use of a combination of longitudinal and cross-sectional collections, these are only an indication of Hh prevalence.

**Table 1:** Source of specimens

Study	Age	Study type	Collection years	Location	Aboriginal or Non-aboriginal	Children in original study (number NTHi +ve)	Number of children NTHi +ve children included in analysis	Specimen type	Number phenotypic NTHi +ve specimens
A^a^	0–6 years	Cross-sectional	2012	Urban	Both	564 (185)	173	Np	172
								ED	1
B [2]	0–3 years	Cross-sectional	2010–2012	Remote	Aboriginal	444 (328)	313	Np	308
								ED	5
C [2]	5–8 years	Cross-sectional	2010–2012	Remote	Aboriginal	138 (133)	80	N	78
								ED	4
D [10]	0–7 months	RCT cohort	2011–2013	Remote	Aboriginal	63 (57)	57	Np	132
								ED	5
E [4]	0–10 years	Cross-sectional	2011–2013	Urban and remote	Both	69 (42)	42	Np	22
								BAL	30
								Throat	16
							Total = 665		Total = 773

*Np* nasopharynx/nasal, *ED* ear discharge, *BAL* bronchoalveolar lavage

^a^Study A: unpublished

#### PCR methods and assays

Samples were prepared for PCR using a colony lysis method; 1–2 colonies from a subculture of each original NTHi isolate were suspended in 200 μl of sterile water, heated at 100 °C for 10 min, cooled on ice, and centrifuged at 12,000 RPM for 2 min to pellet any cellular debris. We used a *Haemophilus* protein D (*hpd*)-based PCR assay employing high resolution melt (PCR-HRM; Rotorgene 6000, Corbett Life Science) to discriminate between NTHi and Hh based upon key single nucleotide polymorphisms as described in detail previously [11]. Briefly, the PCR mix comprised 5 µl of Bioline 2 × SensiMix SYBR Green (QT650-02), 100 nM each of forward and reverse primer, and 1 µl of sample supernatant to a total of 10 µl per reaction. Analysis of the HRM was performed on the Rotorgene software (version 1.7). ATCC strains 19418 and 49274 were used as NTHi controls and ATCC 33390 as the Hh control. Samples were genotyped (NTHi or Hh) according to their melt curve profile relative to the NTHi or Hh controls. All isolates were run in duplicate, and repeated if amplification curves were not within 0.5 cycles of each other. Isolates that did not amplify by *hpd* based PCR were subsequently confirmed as NTHi using a *fucP*-based PCR [12]. PCR assays were performed by laboratory staff who were blinded to the identity, age and location of the participants.

We assessed the percent of phenotypic NTHi isolates genotyped as NTHi, Hh or equivocal (unable to be genotyped), the percent of specimens with had concurrent carriage of both and the percent of specimens requiring reclassification (Table 2). Specimens were reclassified as Hh if all isolates tested (up to four per specimen) were shown to be Hh by PCR-HRM. Data from the five studies were analysed collectively with stratification by the anatomical site of specimen collection. Reclassification isolate and specimen proportions were compared across anatomical sample sites, age group and dwelling (remote versus urban) using the Chi square test (STATA14; StataCorp, USA).

**Table 2:** Proportion of NTHi reclassified as Hh, according to specimen type

Specimen type	No. phenotypic NTHI +ve specimens	No specimens with multiple NTHi scrutinised	No. of phenotypic NTHi isolates tested	No. isolates NTHi by HRM (%)	No. isolates reclassified Hh by HRM (%)	No. isolates equivocal^a^ by HRM (%)	No. specimens with concurrent NTHi and Hh by HRM (%)^b^	No. specimens reclassified Hh by HRM (%)
Np	712	153	879	817 (93)	3 (0.34)	59 (7)	1/153 (1)	2 (0.3)
Ear discharge	15	5	21	21 (100)	0	0	0	0
BAL	30	15	55	34 (62)	14 (25)	7 (13)	7/15 (47)	2 (7)
Throat	16	8	30	15 (50)	12 (40)	3 (10)	2/8 (25)	7 (44)
Total	773	179	985	887 (90)	29 (3)	69 (7)	9/179 (5)	11 (1)

^a^Fail to amplify in PCR-HRM

^b^Denominator no. specimens with multiple NTHi scrutinised

### Results and discussion

Three phenotypic NTHi Np isolates (0.34%, 3/879) and none of the ED isolates (0%, 0/21) were reclassified as Hh by PCR-HRM (Table 2). The three reclassified Hh isolates were from three separate Np specimens, one from each of studies A, C and E. There were too few reclassifications for a valid statistical comparison by the subgroups dwelling and age (Table 3). These very low Hh proportions among Np NTHi isolates are in contrast to an American study [6] of children in day care in which 27.3% of 44 Np NTHi isolates were reclassified as Hh. In a Western Australian (WA) study of 122 children with recurrent otitis media undergoing grommet insertion surgery and 17 healthy controls, (266 isolates in total) 20.5 and 11.8% of Np presumptive NTHi isolates were reclassified by 16s rRNA PCR as Hh, respectively [8]. However only 3.6% (4/110) of otitis prone and 0% of healthy control Np specimens were reclassified as having Hh-only. Differences in results could be real, or accounted for by multiple factors, such as geographic location, season, age, ethnicity, disease severity and density of Np colonisation, or speciation methodology. All studies included children < 36 months. Neither ethnicity nor remoteness was reported in the WA or American cohorts. The Indigenous children in our studies (B, C and D) are at high risk of otitis media and have NTHi Np carriage rates of over 70% for most of their early childhood [2]. In this population the Hh may not be competitive, and therefore absent or not detected, in the Np.

**Table 3:** Reclassification of isolates and specimens as Hh by the subgroups age and dwelling

Specimen type	Subgroup		Hh isolate reclassification, n (%)	p value	Hh specimen reclassification, n (%)	p value
Np	Age^a^	≤1	0/275 (0)		0/218 (0)	
Isolates, n = 879		>1–3	1/413 (0.2)	0.414	0/334 (0)	NA
Specimen, n = 712		>3–10	2/191 (1)	0.089	2/160 (1)	0.098
	Dwelling	Urban	1/193 (0.2)	0.633	1/174 (0.6)	0.400
		Remote	2/686 (0.3)		1/538 (0.2)	
BAL	Age	≤1	–			
Isolates, n = 55		>1–3	13/44 (30)	0.164	2/24 (8)	0.464
Specimen, n = 30		>3–10	1/11 (9)		0/6 (0)	
	Dwelling	Urban	5/10 (50)	0.049	0/4 (0)	0.556
		Remote	9/45 (20)		2/26 (8)	
Throat	Age	≤1	–	–		
Isolate, n = 30		>1–3	8/23 (35)	0.290	4/11 (36)	0.377
Specimen, n = 16		>3–10	4/7 (57)		3/5 (60)	
	Dwelling	Urban	5/6 (83)	0.015	2/3 (66)	0.375
		Remote	7/24 (29)		5/13(38)	

^a^Age p values referenced to ≤ 1

In contrast to the Np, significantly more BAL (25%, 14/55, p < 0.001) and throat (40%, 12/30; p < 0.001) isolates were reclassified as Hh. Throat specimens were less likely to be mixed (NTHi and Hh) and significantly more likely than BAL (7/16, 44% versus 2/30, 7%) to be reclassified as Hh-only (p = 0.003). These specimens were collected from children with a history of lung disease, and the higher Hh proportion in BAL specimens is consistent with that described in American adults with chronic obstructive pulmonary disease (39.5% of sputum isolates were Hh) [6]. For both BAL and throat specimens there was no significant difference in Hh prevalence by age group (BAL p = 0.464; throat p = 0.377), although there were no BAL nor throat samples from children aged ≤ 1 year. In contrast to Np data, NTHi isolates from both the BAL and throat of urban children were significantly more likely be to be reclassified as Hh compared to remote children, p = 0.049 and 0.015 respectively (Table 3). This did not remain significant at the specimen level (BAL p = 0.556; throat p = 0.375), due to high proportion of BAL and throat specimens with both NTHi and Hh.

A small proportion (69/985, 7%) of isolates from Np, BAL and throat specimens failed to amplify in the *hpd*-based PCR-HRM (Table 2). This was presumably due to considerable variation or absence of the *hpd* gene as shown previously [13]. In one study up to 13% of Np and disease-related NTHi isolates were shown to be missing the *hpd* gene by whole genome sequencing [13]. This has potential implications for the efficacy of the pneumococcal *H. influenzae* protein D conjugate vaccine (PHiD-CV10) against *H. influenzae*, and for *hpd*-based diagnostics.

In conclusion, Hh prevalence varied markedly among respiratory specimen types. Our data support a recommendation for DNA-based discrimination of NTHi and Hh for throat and BAL isolates, however, the low prevalence of Hh in nasal or nasopharyngeal swabs (0.34% of presumptive NTHi) suggests that PCR discrimination is not routinely required for these specimens in this population. As the prevalence of Hh in other settings differs, population-specific recommendations for discrimination of Hh and NTHi are required to accurately determine the burden of disease attributable to NTHi. Multiple colony testing is recommended for throat and lung specimens as mixed NTHi and Hh populations can be expected.

## Limitations

We have identified a wide variation in Hh prevalence between our studies and those reported. Due to low rates of positivity we do not recommend *hpd* based PCR methods for discrimination of NTHi and Hh in the Np in this population. Larger studies should be conducted to clarify best application of PCR methods for clinical specimens from the middle ear, lung and throat. It is unclear why Hh reclassification was concordant among lung specimens across studies, yet discordant among nasopharyngeal specimens. Differences in methodology (16S rRNA PCR [6, 8] in combination with MLST and DNA hybridisation [6], PCR-HRM [11]) may have contributed. Geographic location, remoteness of dwelling, variation in NTHi carriage and density and ethnicity could also contribute to this difference.

## Abbreviations

NTHi: non-typeable *Haemophilus influenzae*
Hh: *Haemophilus haemolyticus*
Np: nasopharynx/nasal
ED: ear discharge
BAL: bronchoalveolar lavage
PCR: polymerase chain reaction
DNA: deoxyribonucleic acid
NT: Northern Territory
PCR-HRM: polymerase chain reaction high resolution melt
MLST: multi locus sequence testing
